# Performance measurement of intraoperative systolic arterial pressure to predict in-hospital mortality in adult liver transplantation

**DOI:** 10.1038/s41598-017-07664-0

**Published:** 2017-08-01

**Authors:** Hyung-Chul Lee, Ho-Geol Ryu, Chul-Woo Jung

**Affiliations:** Department of Anesthesiology and Pain Medicine, Seoul National University College of Medicine, Seoul National University Hospital, Seoul, Republic of Korea

## Abstract

Profound hypotension during liver transplantation is aggressively treated with vasopressors thus frequently unrevealed in a retrospective study. The relationship between concealed intraoperative hypotension and in-hospital mortality after liver transplantation was evaluated using performance measurement (PM) of systolic arterial pressure (SAP). Median performance error (MDPE), median absolute performance error (MDAPE), and wobble of SAP were calculated using preoperative SAP as the reference value, and prereperfusion and postreperfusion SAPs as measured values. Univariable and multivariable logistic regression analyses were performed using 6 PM parameters and 36 traditional SAP-derived parameters to predict in-hospital mortality. In-hospital mortality was 3.9% (22/569 cases). Prereperfusion MDAPE and postreperfusion wobble were the only significant SAP-derived predictors of in-hospital mortality. The area under receiver operating characteristic curve of prediction model was 0.769 (95% confidence interval 0.732–0.803, *P* < 0.001; sensitivity = 55%, specificity = 94%). Severe hypotension during liver transplantation is concealed by proactive vasopressor treatment thus traditional measures of hypotension generally fail to detect the masked hypotension in retrospective analysis. PM analysis of intraoperative SAP including prereperfusion MDAPE and postreperfusion wobble is most likely to detect treated and therefore concealed hypotension, and was able to independently and quantitatively predict in-hospital mortality after liver transplantation with high diagnostic specificity.

## Introduction

Patients with advanced liver disease receiving liver transplantation often become hypotensive during surgery. Factors that promote hypotension include profound systemic vasodilation^1^, negative inotropy and vasodilation associated with anesthetics^2^, potential for excessive bleeding during hepatectomy, and postreperfusion syndrome (PRS) after reperfusion of the liver graft^3^. Contrary to common belief and practice, detailed analysis of intraoperative blood pressure showed that well-maintained average blood pressure does not always correlate with improved outcome^4^. The *average* blood pressure is less likely to distinguish between stable blood pressure and multiple episodes of hypotension followed by rapid correction, which is common during liver transplantation.

Performance measurement (PM) is a set of quantitative measures used in pharmacokinetic research that evaluates the discrepancy between the target or predicted concentration and the measured concentration^5^. In this retrospective study, PM parameters were adapted to measure bias, accuracy, and time-dependent variability in blood pressure during liver transplantation surgery by using median performance error (MDPE), median absolute performance error (MDAPE), and wobble, respectively. We hypothesized that PM parameters of systolic arterial pressure (SAP) would more accurately describe the consequences of hypotension during liver transplantation compared to traditional measures such as mean and time-weighted blood pressures.

The primary aim of our study was to evaluate the association between PM parameters of intraoperative SAP and in-hospital mortality after liver transplantation.

## Results

Of the 653 liver transplantation recipients, 569 patients were included in the final analysis. Reasons for exclusion were age less than 20 years old (63 patients), retransplantation (9 patients), intraoperative cardiac arrest (4 patients), and incomplete patient data (5 patients). Two non-survivors who died from non-medical causes were also excluded.

In-hospital mortality rate was 3.9% (22/569 patients). The causes of deaths were sepsis and multi-organ failure (14 patients), postoperative bleeding and associated cardiac events (3 patients), acute myocardial infarction (2 patients), pneumonia (1 patients), irreversible brain damage (1 patient) and unknown (1 patient).

Patient characteristics are described in Table 1. Non-survivors were associated with older age, higher model for end-stage liver disease (MELD) score, more bleeding and transfusion amount, more frequent use of vasopressors, and longer intensive care unit (ICU) length of stay compared to survivors.

**Table 1:** Patient characteristics.

	Survivors (n = 547)	Non-survivors (n = 22)	*P*-value
**Basic characteristics**			
Age (years)	54 (49–60)	60 (54–68)	0.009
Gender (Male)	391 (71%)	13 (59%)	0.172
BMI (kg m^−2^)	23.2 (21.1–25.6)	23.7 (21.5–25.9)	0.531
MELD score	14 (8–22)	29 (20–36)	<0.001
Diagnosis*			
Liver cirrhosis	465 (85%)	18 (82%)	0.761
Hepatocellular carcinoma	308 (56%)	8 (36%)	0.107
Acute liver failure	32 (6%)	8 (36%)	<0.001
Metabolic disorder	8 (1%)	0 (0%)	1.000
Premedical history			
Hypertension	68 (12%)	3 (14%)	0.747
Diabetes mellitus	131 (24%)	6 (27%)	0.717
Encephalopathy	104 (19%)	6 (27%)	0.405
**Anaesthesia**			
Anaesthesia time (min)	465 (400–528)	445 (360–545)	0.498
Estimated blood loss (mL)	2860 (1660–5545)	6073 (3000–8275)	0.004
**Transfused blood components**			
Red blood cell (units)	6 (2–12)	10 (6–14)	0.029
Fresh frozen plasma (units)	6 (0–12)	10 (6–12)	0.012
Platelet concentrate (units)	0 (0–6)	6 (0–6)	0.015
Administered volume (mL)	3500 (2300–5000)	4250 (2500–5200)	0.239
**Use of drugs**			
Dopamine infusion	60 (11%)	8 (36%)	0.002
Norepinephrine infusion	17 (3%)	7 (32%)	<0.001
Epinephrine infusion	8 (1%)	2 (9%)	0.053
Ephedrine (mg)	20 (10–30)	20 (5–25)	0.298
Epinephrine (mcg)	0 (0–20)	15 (0–60)	0.039
**Length of postoperative stay**			
ICU stay (days)	5 (4–6)	17 (8–54)	<0.001
Hospital stay (days)	15 (12–21)	35 (10–64)	0.112

Data are expressed as median (interquartile range) or numbers (%). Characteristics of two groups were compared with Pearson chi-square test or Fisher’s exact test and Mann-Whitney test. *The sum of diagnoses exceeds the number of patients in each group because a part of patients have complex disease entities. Abbreviations: ICU = intensive care unit; MELD = model for end-stage liver disease.

Reference SAP (median [interquartile range]) was not different between the survivors and non-survivors (105 [104–107] vs 103 [93–111], *P* = 0.442). A total of 43,081 intraoperative SAPs (73 [62–87] per patient) were analysed. The number of SAP measurements during the prereperfusion and postreperfusion periods were 39 (28–47) and 36 (29–45), respectively.

**Figure 1:**
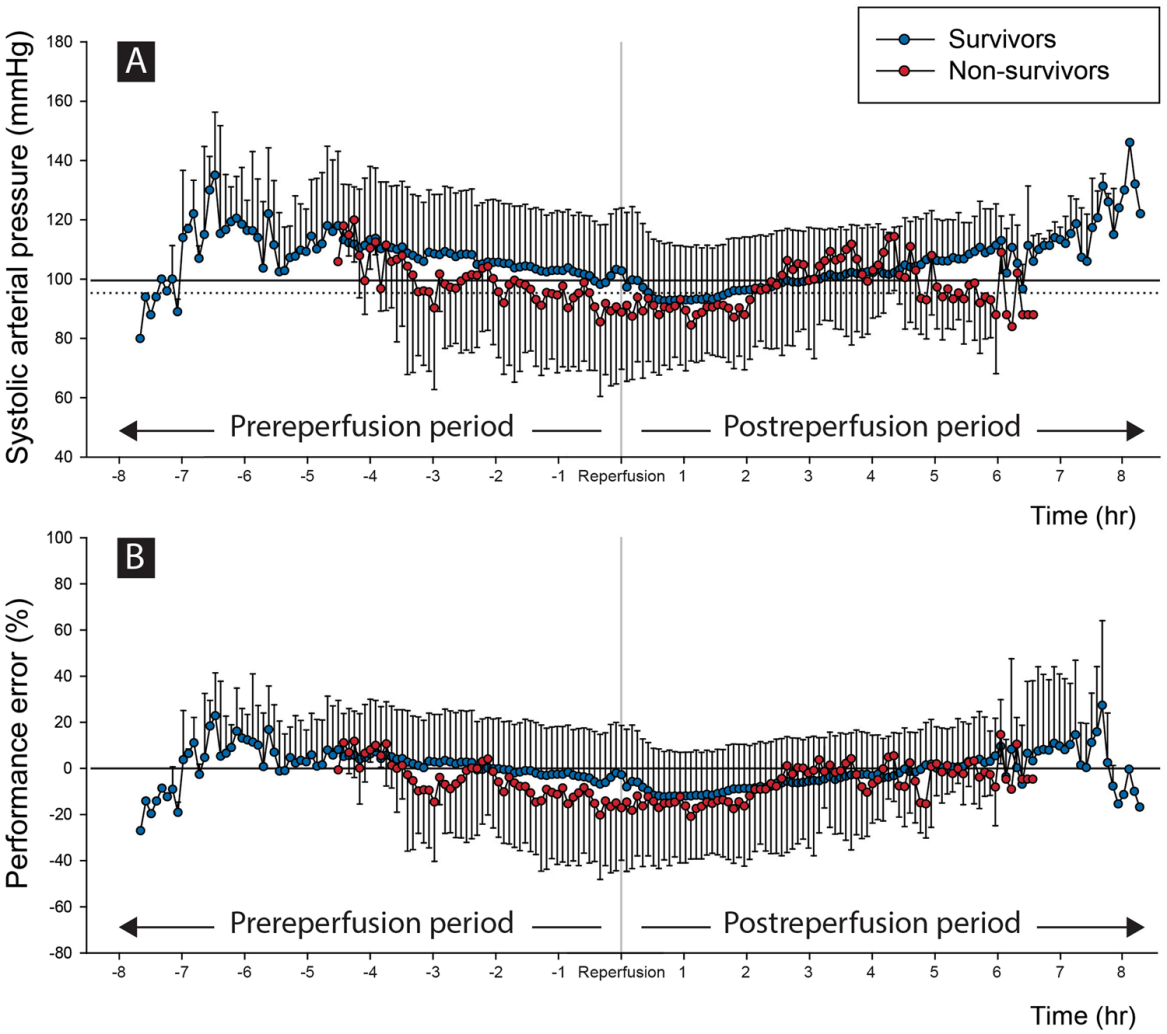
Systolic arterial pressure and performance error during prereperfusion and postreperfusion periods in survivors and non-survivors. Multiple line scatters show the means (dots) and standard deviations (error bars) of survivors and non-survivors. Horizontal lines are averages of intraoperative systolic arterial pressure in survivors (solid line) and non-survivors (dashed line) in Fig. 1A. The graphs start from the time of reperfusion then go towards the start or end of surgery, thus both ends of plot involves only a small number of patients. On average, systolic arterial pressures are a well-maintained around the average systolic arterial pressure in both the survivors and non-survivors during surgery (Fig. 1A). However, performance errors of non-survivors are more deviated and fluctuate above and below the target 0% level, compared to those of survivors (Fig. 1B).

SAP and performance error (PE) of survivors and non-survivors during the prereperfusion and postreperfusion periods are illustrated in Fig. 1. SAPs are maintained around the average SAP in both the survivors and non-survivors. However, PEs of non-survivors tend to be more deviated and fluctuate above and below 0% level compared to survivors.

**Figure 2:**
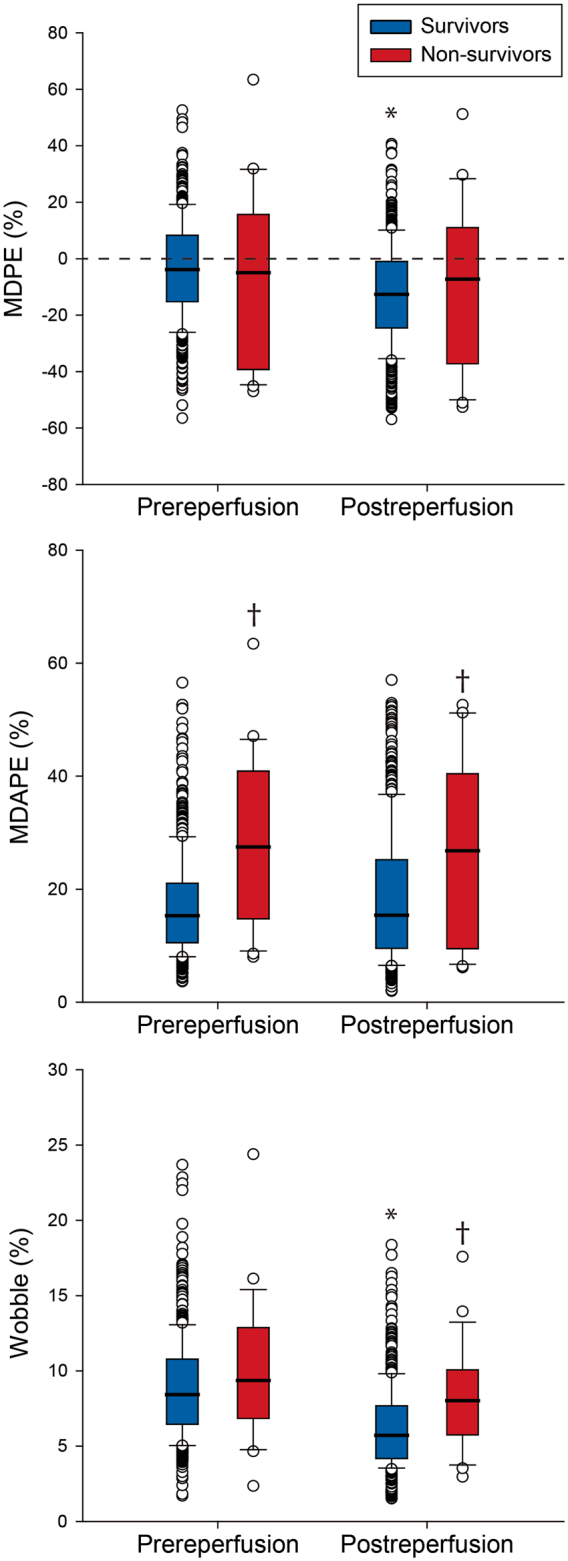
Comparison of performance measures between survivors and non-survivors. Box plot shows median and interquartile range of prereperfusion and postreperfusion MDPE, MDAPE, and wobble in survivors and non-survivors. Upper and lower whiskers are maximum and minimum values, respectively. Round symbols show the 5th/95th percentile values. Prereperfusion and postreperfusion MDPE were similar between groups. However, prereperfusion and postreperfusion MDAPE, and postreperfusion wobble were significantly larger in non-survivors compared to survivors. *P < 0.001 vs prereperfusion values, ^†^ *P* < 0.001 vs survivors. Abbreviations: MDPE = median performance error, MDAPE = median absolute performance error.

PM parameters of SAP in survivors and non-survivors are shown in Fig. 2. Prereperfusion and postreperfusion MDPE were similar between survivors and non-survivors. However, prereperfusion and postreperfusion MDAPE, and postreperfusion wobble were significantly larger in non-survivors compared to survivors (*P* < 0.05).

Table 2 compares traditional SAP parameters between prereperfusion and postreperfusion periods and between survivors and non-survivors. Survivors were more hypotensive during the postreperfusion period compared to the prereperfusion period but with smaller range, interquartile range and standard deviation. In non-survivors, postreperfusion range and standard deviation were significantly larger than prereperfusion values. When non-survivors were compared with survivors, areas <60 mmHg and <40% below reference SAP during prereperfusion period, and range, interquartile range and standard deviation during postreperfusion period were significantly larger in non-survivors.

**Table 2:** Traditional variables of systolic arterial pressure.

	Survivors (n = 547)		Non-survivors (n = 22)	
	Prereperfusion	Postreperfusion	Prereperfusion	Postreperfusion
Mean (mmHg)	104 (94–113)	95 (84–104)*	98 (86–111)	93 (80–109)
Median (mmHg)	102 (92–113)	94 (84–103)*	98 (85–106)	92 (70–109)
Minimum (mmHg)	70 (60–82)	72 (58–84)	67 (50–76)	70 (50–80)
Maximum (mmHg)	140 (126–152)	120 (108–134)*	138 (120–144)	119 (106–144)
25th percentile (mmHg)	94 (84–103)	88 (76–96)*	87 (78–96)	86 (66–101)
75th percentile (mmHg)	113 (102–124)	102 (90–110)*	108 (95–124)	103 (94–114)
Range (mmHg)	68 (54–82)	46 (36–66) *	70 (54–86)	59 (42–72)*†
Interquartile range (mmHg)	18 (14–23)	12 (10–16)*	15 (13–20)	19 (15–26)†
Standard deviation (mmHg)	15 (13–19)	10 (8–13)*	15 (13–20)	13 (10–16)*†
Area <100 mmHg (mmHg min)	828 (310–1900)	1325 (520–2840)*	1050 (460–2350)	1700 (510–4015)
Area <90 mmHg (mmHg min)	300 (80–795)	375 (80–1450)*	420 (140–1560)	580 (110–2600)
Area <80 mmHg (mmHg min)	70 (0–280)	60 (0–453)*	130 (23–520)	80 (0–1240)
Area <70 mmHg (mmHg min)	0 (0–70)	0 (0–105)*	30 (0–130)	5 (0–490)
Area <60 mmHg (mmHg min)	0 (0–0)	0 (0–10)*	0 (0–60)†	0 (0–70)
Area <10% below preoperative SAP (mmHg min)	564 (140–1795)	1005 (158–2653)*	746 (158–4172)	775 (35–5283)
Area <20% below preoperative SAP (mmHg min)	180 (16–673)	193 (0–1181)*	190 (7–2598)	250 (0–3090)
Area <30% below preoperative SAP (mmHg min)	22 (0–311)	27 (0–204)*	109 (0–1536)	89 (0–1570)
Area <40% below preoperative SAP (mmHg min)	0 (0–34)	0 (0–58)*	37 (0–454)†	2 (0–422)

Nonparametric statistics are uniformly used because most values fail to pass normality test. Differences between periods and between groups are compared with Wilcoxon test and Mann–Whitney test, respectively. *P < 0.05 *vs* prereperfusion values, ^†^ *P* < 0.05 *vs* survivors. Abbreviation: SAP = systolic arterial pressure.

Univariable logistic regression analysis identified 0 traditional SAP parameters and 3 PM parameters as potential risk factors of in-hospital mortality (Table 3). Multivariable logistic regression analysis identified 2 PM parameters as independent risk factors of in-hospital mortality after liver transplantation.

**Table 3:** Univariable and multivariable logistic regression analyses for in-hospital mortality.

Variables	Univariable analysis		Multivariable analysis	
	Odds ratio (95% C.I.)	*P*-value	Adjusted odds ratio (95% C.I.)	*P*-value
Prereperfusion period				
MDAPE (per%)	1.083 (1.048–1.119)	<0.001	1.091 (1.053–1.131)	<0.001
Postreperfusion period				
MDAPE (per %)	1.045 (1.014–1.077)	0.004		
Wobble (per %)	1.210 (1.075–1.361)	0.002	1.232 (1.091–1.391)	<0.001

Significant risk factors were sequentially identified using univariable (*P* < 0.2) and multivariable (*P* < 0.05) logistic regression analyses. Abbreviations: C.I. = confidence interval; MDAPE = median absolute performance error.

**Figure 3:**
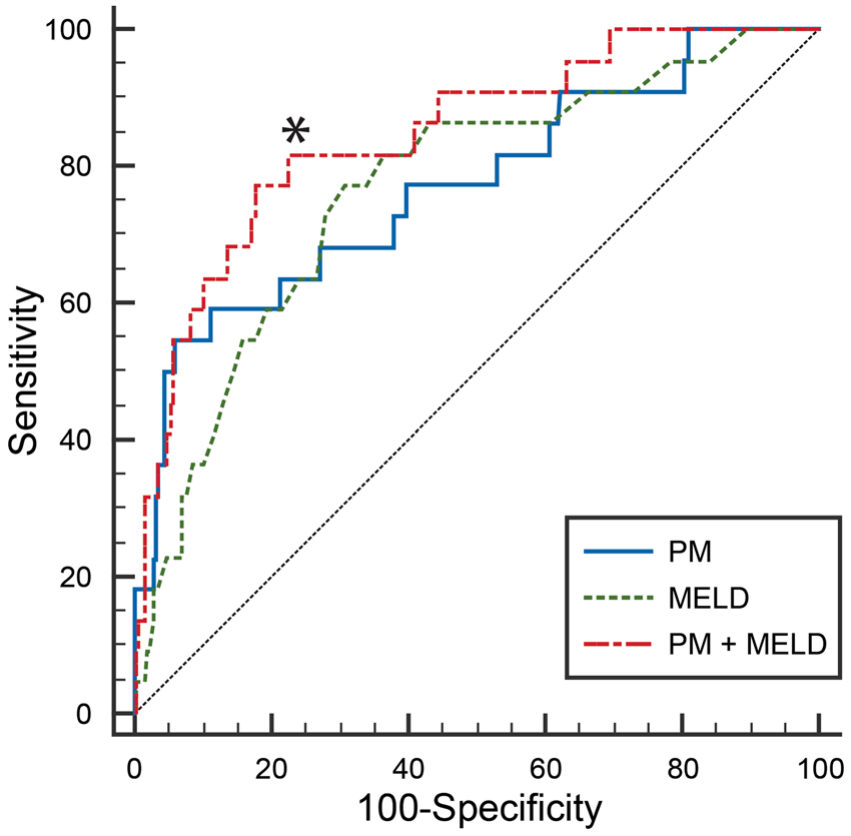
ROC curves of the prediction models. Three ROC curves were built from the propensity scores of prediction models built with performance measures (PM model), MELD score (MELD model) or both (PM + MELD model), respectively. Addition of performance measurement parameters to the MELD model increased the diagnostic specificity of prediction model from 77% to 84%. The combined model has a significantly larger area under ROC curve compared to the PM model. *P = 0.007 vs PM model. Abbreviations: ROC = receiver operating characteristic; PM = performance measurement; MELD = model for end-stage liver disease.

The area under receiver operating characteristic curve (AUROC) of PM model was 0.769 (95% confidence interval [C.I.] 0.732–0.803, *P* < 0.001) with a sensitivity of 55% and a specificity of 94% (Fig. 3). MELD model was also significant with an odds ratio of 1.08 (95% C.I. 1.044–1.116, *P* < 0.001) per point. AUROC of MELD model was 0.767 (95% C.I. 0.730–0.801, *P* < 0.001) with a sensitivity of 77% and a specificity of 69%. The combined PM and MELD model showed increased AUROC (0.847, 95% C.I. 0.815-0.875, *P* < 0.001) with a sensitivity of 77% and a specificity of 82%.

## Discussion

This retrospective study identified that prereperfusion MDAPE and postreperfusion wobble were independent and quantitative predictors of in-hospital mortality after liver transplantation. A prediction model with PM parameters had an intermediate power, low sensitivity, and high specificity in predicting in-hospital mortality after liver transplantation.

Hypotension has been regarded as a cause of morbidity and mortality after surgery. It has been shown that mean arterial pressure (MAP) <60 mmHg for more than 10 min is associated with increased morbidity (odds ratio 5.1) and prolonged hospital stay (odds ratio 4.56) in a prospective observation study of 100 major abdominal surgical procedures^6^. The study claimed that the hypotension itself is associated with tissue hypoperfusion and subsequent adverse effects, regardless of the cause of hypotension. In another prospective observational study that analyzed 1064 patients, every minute of SAP <80 mmHg was associated with increased 1-year postoperative mortality after non-cardiac surgery with an odds ratio of 1.036^7^. However, the underlying cause of hypotension such as hypovolemia, myocardial dysfunction and anesthetic overdose was thought to be more important than hypotension itself. In a retrospective cohort study of 789 liver transplantation recipients, severe hypotension defined as MAP <40 mmHg was associated with unfavorable surgical outcomes including poor graft function, primary nonfunction, or death after surgery^8^. Hypotension was mostly related with the period of liver graft reperfusion, and severe hypotension was suggested as a sign of failed treatment of PRS. PRS has also been related with poor graft outcome, renal dysfunction, longer hospital stay and increased mortality^9–11^. However, despite a number of studies suggesting an association between intraoperative hypotension and postoperative outcomes, intraoperative hypotension has never been well-defined nor well-identified^12^.

The frequency and severity of intraoperative hypotension are usually determined by comparing the intraoperative SAP with a reference SAP, which was largely arbitrary in the literature. Bijker and colleagues^12^ identified 140 different references for defining hypotension in 110 articles published in 4 major anaesthesia journals. Lack of standard definition of hypotension may lead to misunderstanding of outcomes among different situations, misuse of target values in clinical practice and clinical trials, and inappropriate medicolegal conclusions^13^. However, despite the abovementioned concerns, recent studies still adopt a variety of definitions because a single definition of hypotension cannot be applied to various clinical situations^14, 15^. In the current study, the reference SAP to calculate the PE was a relative/individualized SAP rather than an absolute/fixed/non-individualized SAP. Defining intraoperative hypotension based on the individual’s baseline arterial pressure is considered to be a more appropriate assessment of the patient’s status than using fixed or arbitrary threshold values^15^. In addition, the PM parameters show proportional changes relative to the reference level. This enables quantitative assessment of hypotension, which is in contrast to previous dichotomous assessments of hypotensive or not hypotensive. Odds ratios could be calculated per % increment of MDAPE and wobble in the PM model.

The manifestation of hypotension is frequently dependent on patient and surgical characteristics such as age, comorbidity and type and duration of surgery. Therefore, studies of intraoperative hypotension should be tailored to a specific clinical situation with appropriate clinical reasoning. In this study, assessment of PM parameters was designed to be period-dependent because of different underlying mechanisms of hypotension. Hypotension during preperfusion period is usually associated with identifiable causes. It is often a continuum of the preoperative status and is affected by bleeding and volume replacement during hepatectomy, and decreased cardiac preload during clamping of the inferior vena cava after hepatectomy. However, since the mechanism of postreperfusion hypotension is unclear, the treatment is often aimed at hypotension itself, rather than the underlying cause. Postreperfusion hypotension is characterized by the postreperfusion syndrome (PRS), a >30% decrease of mean arterial pressure that occurs within 5 minutes after reperfusion of the liver graft and lasts >1 minute3. Currently significant PRS is also defined as severe haemodynamic instability, persistent hypotension, cardiac dysrhythmia, and development of fibrinolysis requiring pharmacological interventions throughout the postreperfusion period^16^. Treatment involves aggressive use of vasopressors.

Our results show that the degree of hypotension measured by MDPE, mean/median SAP and time-weighted SAP were comparable between survivors and non-survivors. Meanwhile, variability of SAP expressed as MDAPE, wobble, range of SAP and standard deviation were significantly larger in non-survivors, especially during the postreperfusion period. We speculate that the occurrence of profound hypotension was masked by proactive vasopressor treatment resulting in a well maintained average SAP. However, hypotension can still be detected by measuring MDAPE and wobble that calculate absolute deviations of SAPs of both treated and untreated hypotension. Our observation coincides with the report of Milan and colleagues^4^ who showed in their prospective study of 100 liver recipients that non-survivors by 30 days were associated with greater variability of intraoperative MAP compared to survivors. The overall MAP was generally low, but was relatively higher in non-survivors compared to survivors. They assumed that frequent hypotension of non-survivors may have been masked by the use of inotropes resulting in a higher average MAP in non-survivors. Tables 1 and 2 also show that vasopressors were required more often in non-survivors with no difference in the severity of hypotension. In addition to the assertion that both MDAPE and wobble are *traces of hypotension*, we also claim that large postreperfusion wobble may be an expression of *refractory hypotension* to vasopressor treatment. Repeated administration of vasopressors to unresponsive patients may have led to fluctuating SAP measured as a large wobble.

The current study has various limitations. The first limitation to be considered is a matter of data resolution. Vital signs during anaesthesia are traditionally recorded at 5 minute intervals. However, acute changes caused by massive bleeding, volume resuscitation, and administration of potent vasopressors are often hidden in that interval. It has been pointed out that retrospective studies with low resolution data can distort the incidence of PRS, where patients frequently recover from severe hypotension within 5 minutes following appropriate treatment^17^. However, single hypotensive event with immediate recovery is less likely to be associated with adverse outcomes. Consistent and repetitive hypotension is best characterized by MDAPE and wobble that measure cumulative deviation from target value. In addition, the method of PM that deals with median values has been inherently developed to handle sparse data of pharmacokinetic and pharmacodynamic studies. It is less likely that data with shorter interval would show different median values. Nonetheless, the adequate resolution of vital sign data for haemodynamic studies requires further investigation. Second, MAP may seem to be a more suitable reflection of arterial pressure, but SAP rather than MAP was chosen as an independent variable in this study. The main reason was that MAPs are not always measured in the ward preoperatively, and thus a large part of reference values would have been missing if MAP was used. Therefore, SAP is a valid alternate for MAP since the correlation coefficient between SAP and MAP was as high as 0.95 ± 0.04 in our preliminary correlation analysis using all intraoperative data of current study. In addition, the most frequently investigated variable in studies of hypotension has been the SAP^12^. Third, the PM model has a low sensitivity, thus is less likely to be useful in screening in-hospital mortality. However, the addition of PM may increase the diagnostic specificity of a test with low predictability. The MELD score, which was not originally designed to predict postoperative outcome in liver recipients, has been reported to be a weak predictor of posttransplant mortality^18^. But, postsurgical mortality of liver recipients was well predicted by MELD Model combined with donor or recipient factor like Muscle-MELD or D-MELD^19, 20^. Our result shows that intraoperative hypotension measured by PM was a clinically relevant risk factor that improves the predictability of MELD model. Fourth, it should be noted that the diagnosis of acute liver failure (ALF) was common in non-survivors. Although there is no definitive method for predicting mortality in ALF patients, the degree of accompanied encephalopathy is known to be a risk factor^21^. In contrast to chronic liver failure patients, ALF patients frequently show acute and severe neurologic deterioration, which may be associated with post-transplant mortality. Fifth, haemodynamic evaluation using PM may be considered to be of limited clinical value. However, since the PM can be continuously calculated using intraoperative SAPs, the postoperative mortality rate can be quantitatively reduced through interventions aimed at improving the PM value. The effectiveness of PM-guided management should be evaluated in future prospective randomised trials. Final limitation is the issue with artifacts that is almost inevitable in retrospective studies. The reported incidence of artifacts in invasive blood pressure in a prospectively study of vital sign data obtained from the anaesthesia information management system was as high as 14%^22^. Most of the artifacts in invasive blood pressure measurement come from simple errors such as misplaced transducers or damping of the pressure system. However, it is difficult to differentiate artifacts from real hypotension in data that has been collected retrospectively. Nonetheless, we believe that the rigorous monitoring during liver transplantation surgery, low resolution of anaesthesia records, and use of PM may have contributed to minimizing artifacts.

In conclusion, intraoperative hypotension during liver transplantation shows characteristic features during the prereperfusion and postreperfusion periods. Severe hypotension in these periods is frequently concealed by proactive vasopressor treatment, thus traditional measures of hypotension generally fail to detect the masked hypotension, especially in retrospective analysis. PM analysis of intraoperative SAP is most likely to detect treated and therefore concealed hypotension, and was able to independently and quantitatively predict in-hospital mortality after liver transplantation. We propose that PM is a useful method to quantify the degree of hypotension during liver transplantation. In addition, combining PM parameters with other risk factors may significantly boost the diagnostic power of a prediction model.

## Methods

The study protocol was approved by the institutional review board of Seoul National University Hospital (H-1107-110-371). Informed consent was waived due to the retrospective study design. Acquisition of living or deceased liver grafts was performed at the Hospital Based Organ Procurement Organization (HOPO) designated by law, and none of grafts were obtained from the executed prisoners.

### Patient selection

Patients who underwent liver transplantation between January 2012 and December 2015 were screened for eligibility. Paediatric cases (19 years or younger), former records of re-transplantation, and patients with incomplete anaesthesia record were excluded.

### Anaesthesia and headmodynamic management

Anaesthesia was performed according to the liver transplantation anaesthesia protocol of our institution during the study period. Electrocardiography, non-invasive blood pressure, and pulse oximetry monitoring were applied. Anaesthesia was induced with 1.5–2 mg kg^−1^ of propofol, 0.9–1.2 mg kg^−1^ of rocuronium and sevoflurane with oxygen. Volume controlled ventilation was maintained with a tidal volume of 6–8 mL kg^−1^, a frequency of 10–15 min^−1^, and an F_I_O_2_ of 0.5. Anaesthesia was maintained with sevoflurane and continuous infusion of rocuronium. Arterial lines were inserted into the radial and femoral arteries. The radial arterial line was used for frequent blood sampling and the femoral arterial line was used for uninterrupted blood pressure monitoring. Triple lumen Swan-Ganz introducer and pulmonary artery catheter were placed into the right internal jugular vein. Anaesthetic management during prereperfusion period was focused on maintaining cardiac preload by monitoring the central venous pressure and cardiac stroke volume. Intermittent bolus of ephedrine was our first choice of treatment for hypotension during prereperfusion period, but epinephrine or dopamine were administered to the patients who showed persistent hypotension. Immediately after reperfusion, initial hypotension was treated with 10 mg of ephedrine, but acute and severe hypotension (SAP <60 mmHg or MAP <40 mmHg within 1 minute after reperfusion) was treated with 10 mcg of epinephrine. If the severe hypotension continued, repeated doses of epinephrine were administered every minute until MAP ceased to decrease or started to increase. Continued hypotension after 5 repeated doses of epinephrine required titrated infusion of dopamine or norepinephrine to recover at least 70% level of the baseline SAP or MAP.

### SAP-derived parameters

Reference SAP was defined as the median of 5 consecutive SAPs measured before surgery. Preoperative SAP was obtained from the electronic medical chart during the preoperative period in the ward or the ICU. A portable non-invasive blood pressure monitoring device was used at ward, however an invasive arterial pressure monitoring was applied to the ICU patients. The recent 5 consecutive SAP, measured at an interval of at least 4 hours, were collected and the median value was used for the PM calculation.

Intraoperative SAP was retrieved from the electronic anaesthesia records, where SAP values were automatically recorded every 5 minutes. All SAP data were classified into the prereperfusion period (from 10 min after the start of surgery to the reperfusion of the liver graft) or the postreperfusion period (from the time of reperfusion to the end of surgery). SAP-derived variables were calculated separately according to the reperfusion periods.

#### Performance measurement of intraoperative SAP

The performance of intraoperative SAP was calculated using the method of Varvel and colleagues^5^. The formula was originally proposed to describe the discrepancy between the predicted and measured drug concentrations in pharmacokinetics, and in recent studies, has also been used to evaluate how well target SAP or BIS is achieved during anaesthesia^23, 24^. The predicted and measured drug concentrations in the original formula were substituted with the reference and measured SAPs in the current study.

The first step of PM was to calculate PE at every time point.

$${\rm{PE}}\,( \% )=(\mathrm{measured}\,\mathrm{SAP}-\mathrm{reference}\,\mathrm{SAP})\times 100/{\rm{reference}}\,{\rm{SAP}}$$

MDPE and MDAPE were calculated by the following formulas.

$${\rm{MDPE}}\,( \% )={\rm{median}}\{{{\rm{PE}}}_{{i}},i=1,2,3\ldots ,\,{\rm{N}}\}$$

$${\rm{MDAPE}}\,( \% )={\rm{median}}\{{|{\rm{PE}}|}_{{i}},{\rm{i}}=1,2,3\ldots ,\,{\rm{N}}\}\quad ({\rm{N}},\,{\rm{number}}\,{\rm{of}}\,{\rm{measured}}\,{\rm{SAP}})$$

Wobble is the median absolute value of the differences between MDPE and PE for the measured period, and expressed by the following formula.

$${\rm{Wobble}}\,( \% )={\rm{median}}\{|{\rm{MDPE}}-{{\rm{PE}}}_{{i}}|,{i}=1,2,3\ldots ,\,{\rm{N}}\}({\rm{N}},\,{\rm{number}}\,{\rm{of}}\,{\rm{measured}}\,{\rm{PE}})$$

From the SAP perspective, negative MDPE can be interpreted as relative hypotension. Increased MDAPE implies a large difference between measured SAP and the reference SAP, but does not distinguish between hypertension and hypotension. A large wobble indicates unstable SAP, which manifests fluctuation of SAP below and above the average SAP. If all 3 PM parameters were near 0%, it can be considered to be a successful haemodynamic management.

PM parameters were calculated for both prereperfusion and postreperfusion periods (6 parameters) using the Excel® program (*version* 2013, Microsoft Corporation, WA).

#### Traditional variables of intraoperative SAP

Traditional SAP parameters were calculated for prereperfusion and postreperfusion periods. The parameters included mean, median, minimum, maximum, 25th and 75th quartiles, range, interquartile range and standard deviation of SAP. To account for both the duration and severity of hypotension, the area under threshold values were calculated by multiplying the threshold value by the duration under the threshold value. Area under 100, 90, 80, 70 and 60 mmHg, and 10, 20, 30 and 40% of preoperative SAP were calculated. A total of 36 traditional SAP-derived variables were calculated.

### Statistical analysis

Current study data can be found in Supplement 1.

Characteristics of survivors and non-survivors were compared with Pearson chi-square test or Fisher’s exact test and Mann-Whitney test. In-group and between-group differences of SAP-derived variables were evaluated with Wilcoxon test and Mann-Whitney test, respectively.

Univariable logistic regression analyses were performed with 6 PM parameters and 36 traditional SAP-derived parameters as independent risk factors and in-hospital mortality as the dependent outcome. Thereafter, a multivariable logistic regression analysis with forward selection method was performed using variables selected in the univariable analyses (*P* < 0.2). The predicted risks of individual patients were calculated with the prediction model, followed by the receiver operating characteristic curve analysis using the predicted risks as the independent variable and in-hospital mortality as the classification variable. AUROC was calculated to evaluate the diagnostic power of the model.

In addition, traditional risk factor such as MELD score was tested with univariable and multivariable logistic regression. The AUROC of SAP-derived parameters model, MELD model and combined model were compared.

Data are expressed as median (interquartile) or absolute number. Statistical analyses were performed using the SPSS software (*version* 21, SPSS Inc., IL, USA) and MedCalc software (version 16.8.4, MedCalc Software bvba, Ostend, Belgium). *P*-values less than 0.05 were considered statistically significant.

## Electronic supplementary material

Supplementary Dataset
